# Combined exercise and nutrition intervention for older women with spinal sarcopenia: an open-label single-arm trial

**DOI:** 10.1186/s12877-023-04063-1

**Published:** 2023-06-01

**Authors:** Seungcheol Kim, Jinhee Park, Dong Hyun Kim, Jiyu Sun, Sang Yoon Lee

**Affiliations:** 1 Department of Rehabilitation Medicine, Seoul National University College of Medicine, Seoul National University Hospital, Seoul, Republic of Korea; 2 Department of Rehabilitation Medicine, Seoul National University College of Medicine, SMG-SNU Boramae Medical Center, 20 Boramae-ro 5-gil, Dongjak-gu, Seoul, 07061 Republic of Korea; 3 Department of Radiology, Seoul National University College of Medicine, SMG-SNU Boramae Medical Center, Seoul, Republic of Korea; 4 Integrated Biostatistics Branch, Division of Cancer Data Science, National Cancer Center, Goyang-si, Korea

**Keywords:** Nutritional support, Paraspinal muscles, Resistance Training, Sarcopenia, Spine

## Abstract

**Purpose:**

Spinal sarcopenia is a multifactorial disorder associated with atrophy and fatty changes in paraspinal muscles. Interventional studies for spinal sarcopenia are limited. We aimed to evaluate the effectiveness of a combined exercise and nutrition intervention for the treatment of spinal sarcopenia.

**Methods:**

35 community-dwelling older women diagnosed with spinal sarcopenia in a previous cohort study were included. The 12-week combined intervention consisted of back extensor strengthening exercises and protein supplementation. The following outcomes were measured at baseline (week 0), after the intervention (week 12), and follow-up (week 24): conventional variables of sarcopenia (appendicular skeletal muscle mass, handgrip strength, 6-meter gait speed, and short physical performance battery); lumbar extensor muscle mass; lumbar extensor muscle volume and signal intensity; back extensor isokinetic strength; and back performance scale. We used the intention-to-treat analysis method, and repeated measures analysis of variance was used to analyze the data.

**Results:**

Of the total 35 potential participants, 26 older women participated in the study (mean age 72.5 ± 4.0 years old). After 12 weeks of combined exercise and nutrition intervention, there were no changes in the appendicular skeletal muscle mass, lumbar extensor muscle mass, volume, or signal intensity. Handgrip strength and back extensor isokinetic strength did not change significantly. Short physical performance battery significantly increased (P = 0.042) from 11.46 ± 0.86 to 11.77 ± 0.53 at week 12 and 11.82 ± 0.40 at week 24. The back performance scale sum score also significantly improved (P = 0.034) from 2.68 ± 1.81 to 1.95 ± 1.21 at week 12 and 2.09 ± 1.34 at week 24.

**Conclusion:**

The combined exercise and nutrition intervention for community-dwelling older women with spinal sarcopenia could be feasible and helpful in improving the physical performance as well as back performance.

## Introduction

Spinal sarcopenia is a complex disorder characterized by progressive decline in the paraspinal muscle mass and function. It is characterized by atrophy of paraspinal muscles, with decrease in cross-sectional area and fatty degeneration of the muscles [1]. Loss of paraspinal muscle plays an important role in spinal disorders, such as lower back pain and spinal dysfunction [2]. Paraspinal muscle atrophy is also associated with spinal sagittal imbalance, which is a major parameter for lumbar spinal surgery [3] and non-surgical intervention of spinal stenosis [4].

Conventional sarcopenia variables cannot be used to diagnose spinal sarcopenia. Paraspinal muscle assessment generally requires spinal computed tomography (CT) or magnetic resonance imaging (MRI). However, findings from our spinal sarcopenia-related community-dwelling elderly cohort (SarcoSpine) [1], suggest that the lumbar extensor muscle (LEM) mass measured by lateral whole-body dual-energy X-ray absorptiometry (DXA) is significantly correlated with the results of quantitative analysis of the same region by lumbar spine three dimensional magnetic resonance imaging (L-S spine 3D MRI) [5]. To confirm the function of the paraspinal muscle, we also evaluated variables such as isokinetic or isometric back extensor strength and back performance scale (BPS) [6].

While new drug development is urgently required to treat sarcopenia, there has been no drug from this group which has passed phase III or higher clinical trial testing [7]. Therefore, the current first-line interventions for the prevention and treatment of sarcopenia are still resistance exercise and nutritional supplementation [8, 9]. There is strong evidence for how best to apply progressive resistance exercises, target protein intake, and supply nutrition to older adults with sarcopenia [10, 11]. However, most sarcopenia interventions are focused on extremity muscles that are involved in the classical diagnostic criteria for sarcopenia. Therefore, it is necessary to confirm the feasibility and effectiveness of combined exercise and nutritional interventions for the treatment of spinal sarcopenia.

In this pilot study, we aimed to develop a combined exercise and nutritional intervention for treating spinal sarcopenia in elderly women, and to determine the feasibility and effectiveness of a 12-week period of the combined intervention.

## Materials and methods

### Standard protocol approvals, registrations, and patient consent

The study protocol was approved by the Institutional Review Board of Seoul Metropolitan Government Seoul National University (SMG-SNU) Boramae Medical Center (no. 10-2021-27), and written informed consent was obtained from each participant before the intervention. The study was registered with ClinicalTrials.gov (NCT04810312), and the study protocol has been published [12]. The date of first registration was 22/03/2021.

### Study population

**Fig. 1:**
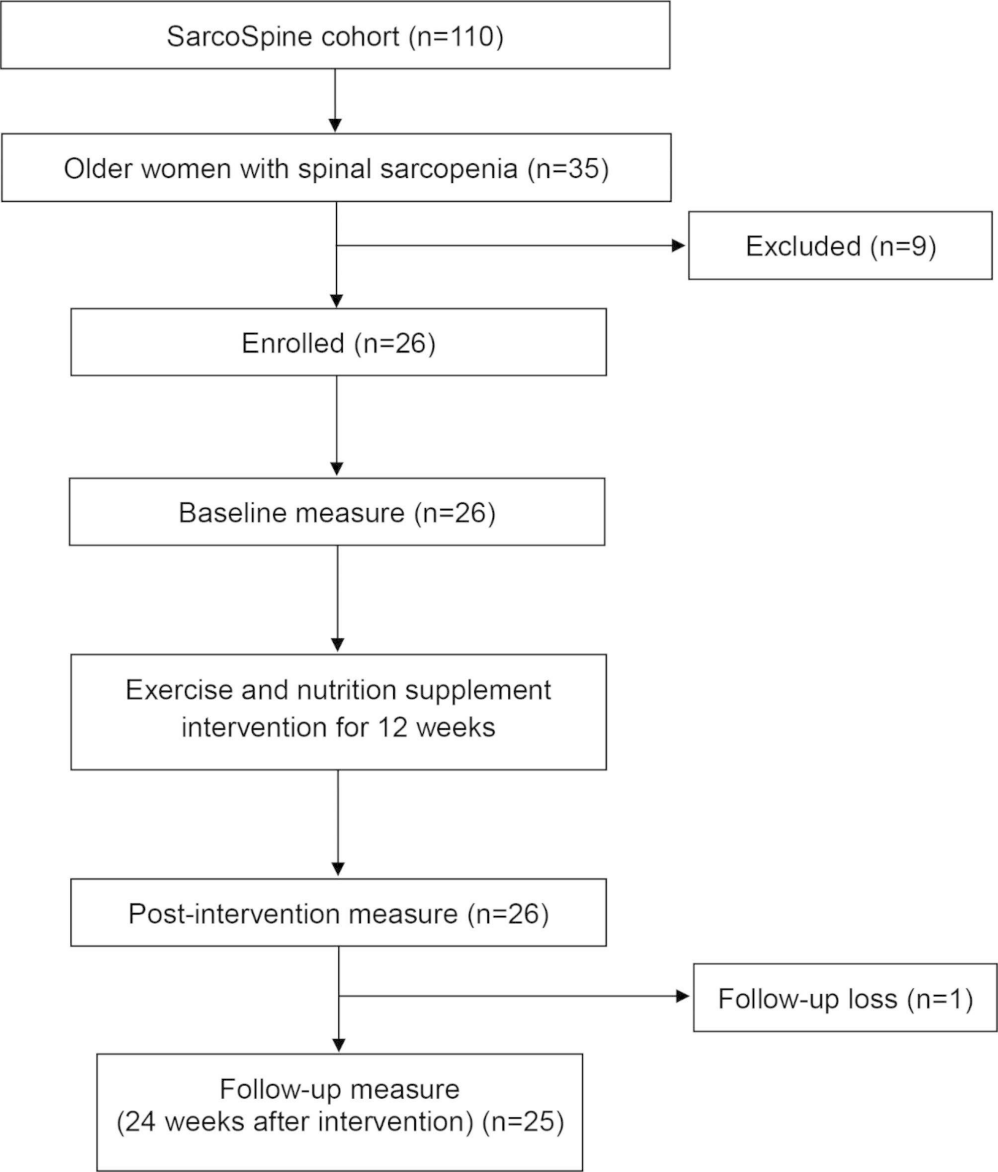
Flow diagram of this study

This study was an open-label, single-arm pilot study conducted in a single center. Eligibility criteria included older (≥ 65 y) community-dwelling women diagnosed with spinal sarcopenia in our previous cohort study (SarcoSpine cohort) [1]. In the cohort study, DXA was performed in the lateral direction to quantitatively evaluate the LEM [5]. The LEM mass were measured for 70 older women in the cohort, the cut-off value of low lumbar extensor muscle mass was 562 g, which belongs to the lower 50th of the measurements in women. Thirty-five female participants with an LEM mass of 562 g or less were defined as having spinal sarcopenia and were eligible for this study. Conventional and spinal variables related to sarcopenia were evaluated at baseline (week 0), after intervention (week 12), and at follow-up (week 24) (Fig. 1).

Individuals who met the following criteria were excluded from this study: (1) lower back pain with a numeric rating scale score of 5 or more; (2) previous surgical history of the lumbar spine; (3) surgical history of hip fracture or hip/knee arthroplasty; (4) contraindications for MRI (e.g., presence of cardiac pacemaker, implanted metallic objects, and claustrophobia); (5) central nervous system disorders (e.g., stroke, parkinsonism, and spinal cord injury); (6) cognitive impairment (mini-mental state examination score < 24); (7) communication disorders (e.g., severe hearing loss); (8) musculoskeletal conditions affecting physical function (e.g., limb amputation); (9) long-term corticosteroid use due to inflammatory disease; (10) malignancy requiring treatment within the previous 5 years; and 11) other medical conditions requiring active treatment [12].

### Combined exercise and nutritional intervention

Participants were divided into groups of two to three individuals, and each group visited our institute biweekly for 12 weeks. In each session, a 50-minute exercise program for spinal extensor strengthening was executed. Exercise intensity was determined by measuring the amount of time (in seconds) that each motion was maintained per set. Before and after each session, 5-minutes of warm up and cool-down on the major muscle groups was included. The back extensor strengthening exercises included (1) 5-minutes of McKenzie back extension exercise; (2) 5-minutes each of curl-up, side-bridge, and bird-dog exercises; (3) 10-minutes of plank exercises (basic > elbow > one-side); and (4) 10-minutes of squatting exercise. An exercise booklet describing the back extensor strengthening exercises was provided to each participant so they could follow the regime of exercising three times per week (including bi-weekly visits to the institution). In the initial evaluation, exercise intensity was individually defined, based on the number of repetitions and maintenance time of each exercise. On their visit to the institution every two weeks, the exercise intensity was increased by 20% so that progressive resistance exercise was possible. Weekly video calls and participation checks were conducted to improve the exercise compliance.

Each participant’s daily calorie and protein intake was calculated using the 24-hour recall method. A liquid protein supplement (125 mL) and an energy bar (Selex core protein; Maeil Health Nutrition Co., Ltd., Gyeonggi-do, Korea; 46 g of carbohydrates, 16 g of fats, and 20 g of proteins with 1830 mg of leucine, providing a total of 423 kcal) were provided to each participant per day for 12 weeks. The supplementation was scheduled to be taken once a day, irrespective of whether it was before or after exercise. To monitor the nutritional supplementation, participants were provided with monitoring sheets to record the time of the day on which they consumed the protein supplement; this data were collected every 2 weeks (institute visiting day) [13]. Compliance with exercise and nutritional intervention was continuously monitored, and a dropout was defined as not reaching 80% compliance at the end of the study. The exercise and nutrition intervention were carried out by a single researcher (JP), while the measurement and analysis of the outcome variables were conducted by the remaining authors, excluding this researcher.

### Outcome measures

Each outcome variable and its measurement method were described in detail in the study protocol paper [12]. The following outcome variables were measured at baseline (week 0), after the intervention (week 12), and at follow-up (week 24).

Back extensor muscle strength was the primary outcome measure. This was measured using an isokinetic dynamometer (Biodex Multi-Joint System; Biodex Corporation, Shirley, NY, USA). Briefly, the examination was performed by comfortably seating the participant in the device, fixing both the legs and back to the machine using a strap. Subjects were instructed to flex and extend their backs five times at an angular velocity of 60°/s as a warm-up before the examination. During the test, subjects were instructed to perform maximum effort back flexion and extension 10 times, with a 60°/s angular velocity. The back range of motion was limited to 50° with 30° (− 30°) flexion and 20° (+ 20°) extension relative to the anatomical reference position (0°) [14]. We measured the peak torque (Nm) and peak torque per body weight (Nm/kg) [15].

Secondary outcome measures were performed as follows [1]:

#### 1. LEM mass, volume, and signal intensity

DXA (Lunar iDXA for Bone Health; GE Healthcare, Schenectady, NY, USA) was used to analyze the LEM mass. A lateral whole-body scan was performed according to the enCORE-based X-ray Bone Densitometer User Manual. After the lateral DXA scan was performed, the region of interest was defined to analyze the LEM composition [5].

**Fig. 2:**
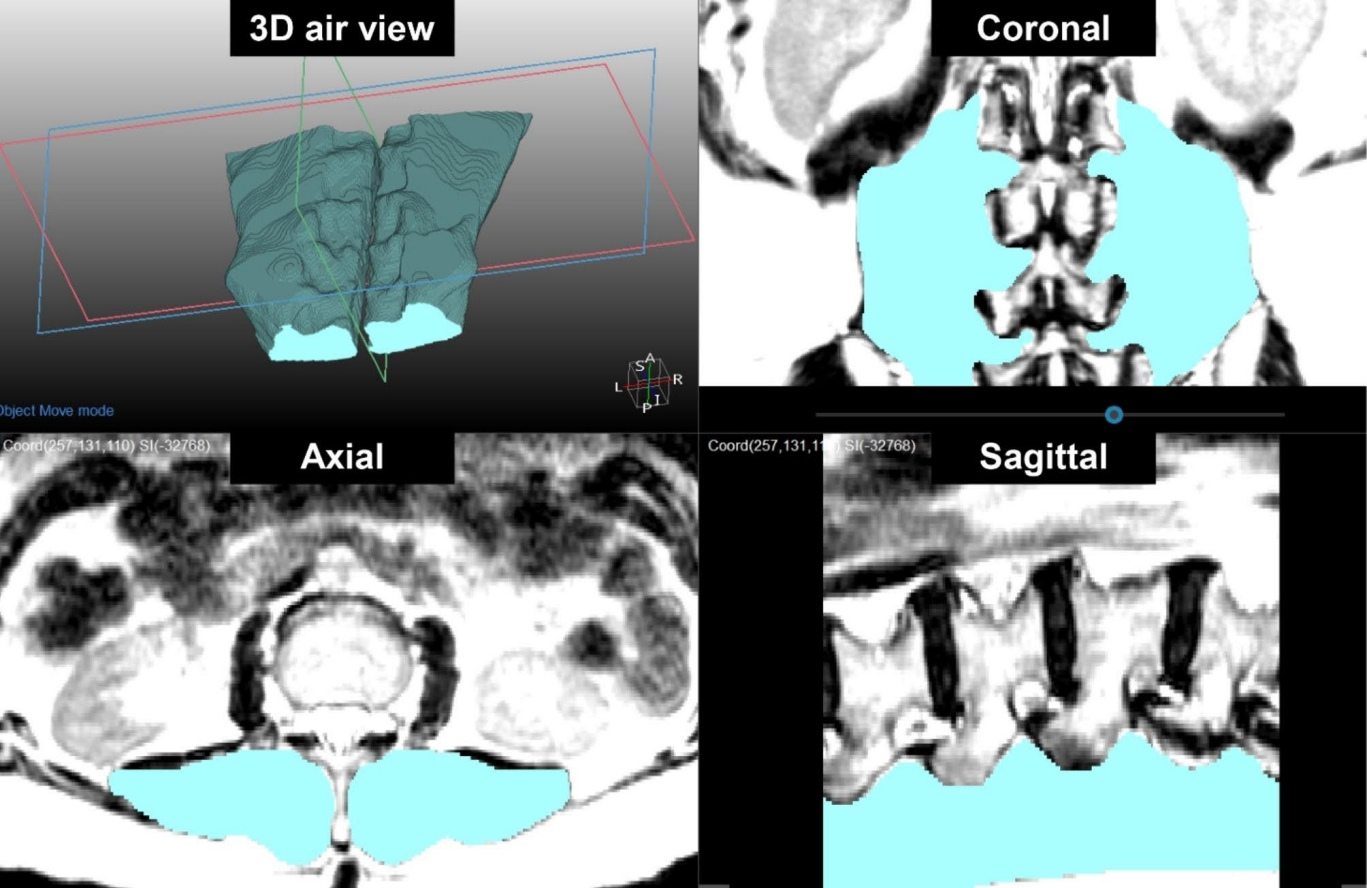
Three-dimensional segmentation of the lumbar extensor muscle

L-S spine 3D-MRI was performed at baseline (week 0) and after the intervention (week 12) using a 1.5-T scanner (Achieva 1.5 T; Philips Healthcare, Netherlands). The imaging protocol included sagittal T2-weighted fast spin-echo imaging and axial T2-weighted fast spin-echo imaging. Axial images (five slices) were obtained for each lumbar intervertebral level (T12/L1 to L4/5) parallel to the vertebral endplates. Three-dimensional segmentation of the LEM was performed to measure the mean volume and signal intensity. The right and left LEM compartments were segmented separately from the mid-disc level of T12/L1 to the mid-disc level of L4/5. A semi-automated random walk 3D segmentation algorithm, a magic cut tool for segmentation within radiological software (MEDIP; MEDICALIP, Seoul, South Korea), was used to volumetrically segment the LEM (Fig. 2) [16]. During segmentation, an experienced radiologist repeatedly modified the procedure and confirmed the segmentation results. In this process, the ROI was positioned at the muscle contour with care taken to avoid accidental inclusion of subcutaneous fat or the muscle-fat interface. The bilateral LEM compartments were combined to determine the mean signal intensity and volume.

#### 2. Back performance scale (BPS)

The BPS is composed of the Sock Test, Pick-up Test, Roll-up Test, Fingertip-to-Floor Test, and Lift Test and is used to assess the trunk mobility and performance. These five performance tests are associated with each other and have high internal consistency, implying that they share the ability to measure the physical performance of the spine [17]. The BPS sum score (0–15) was calculated by adding the individual scores of the five tests.

#### 3. Conventional variables of sarcopenia

DXA was used to analyze the body composition, including the appendicular skeletal muscle mass (ASM), which was calculated by summing the lean masses of the bilateral upper and lower extremities [18], and were standardized by dividing with the squared height value (appendicular skeletal muscle mass/height^2^ (ASM/Ht^2^) [kg/m^2^]). Handgrip strength was measured using a handgrip dynamometer (T.K.K.5401; Takei Scientific Instruments, Tokyo, Japan) [19], as described previously [20, 21]. Gait speed was measured using 6-meter usual gait speed (m/s), as recommended by the Asian Working Group for Sarcopenia [22]. The Short Physical Performance Battery (SPPB), which includes three objective physical function tests (i.e., time taken to cover 4 m at a comfortable walking speed, time taken to stand from a sitting position in a chair five times without stopping, and ability to maintain balance for 10 s in three different foot positions at progressively more challenging levels), was also measured. Subject’s nutritional status was evaluated with body mass index (body weight/height^2^ [kg/m^2^]) and the Mini Nutritional Assessment [23].

### Data analysis and statistics

The intention-to-treat principle was used for data analysis. Participant characteristics are described using means and standard deviations for continuous data. After confirming normality, we used repeated measures ANOVA and paired t-test to compare the paired data between the three time points and two time points, respectively. Statistical significance was defined as a two-sided P-value of < 0.05. All statistical analyses were performed using SAS version 9.4 (SAS Institute, Cary, NC, USA).

## Results

Among the 35 older women with spinal sarcopenia in the SarcoSpine cohort, nine refused to participate. Finally, 26 women were enrolled (mean age 72.5 ± 4.0 years old) (Fig. 1). Baseline (week 0) and post-intervention (week 12) measures were conducted for 26 participants, and follow-up (week 24) measures were performed for 25 participants owing to one loss to follow-up. During the study period, there were no dropouts owing to poor compliance. No adverse events related to exercise-nutrition interventions were reported.

**Fig. 3:**
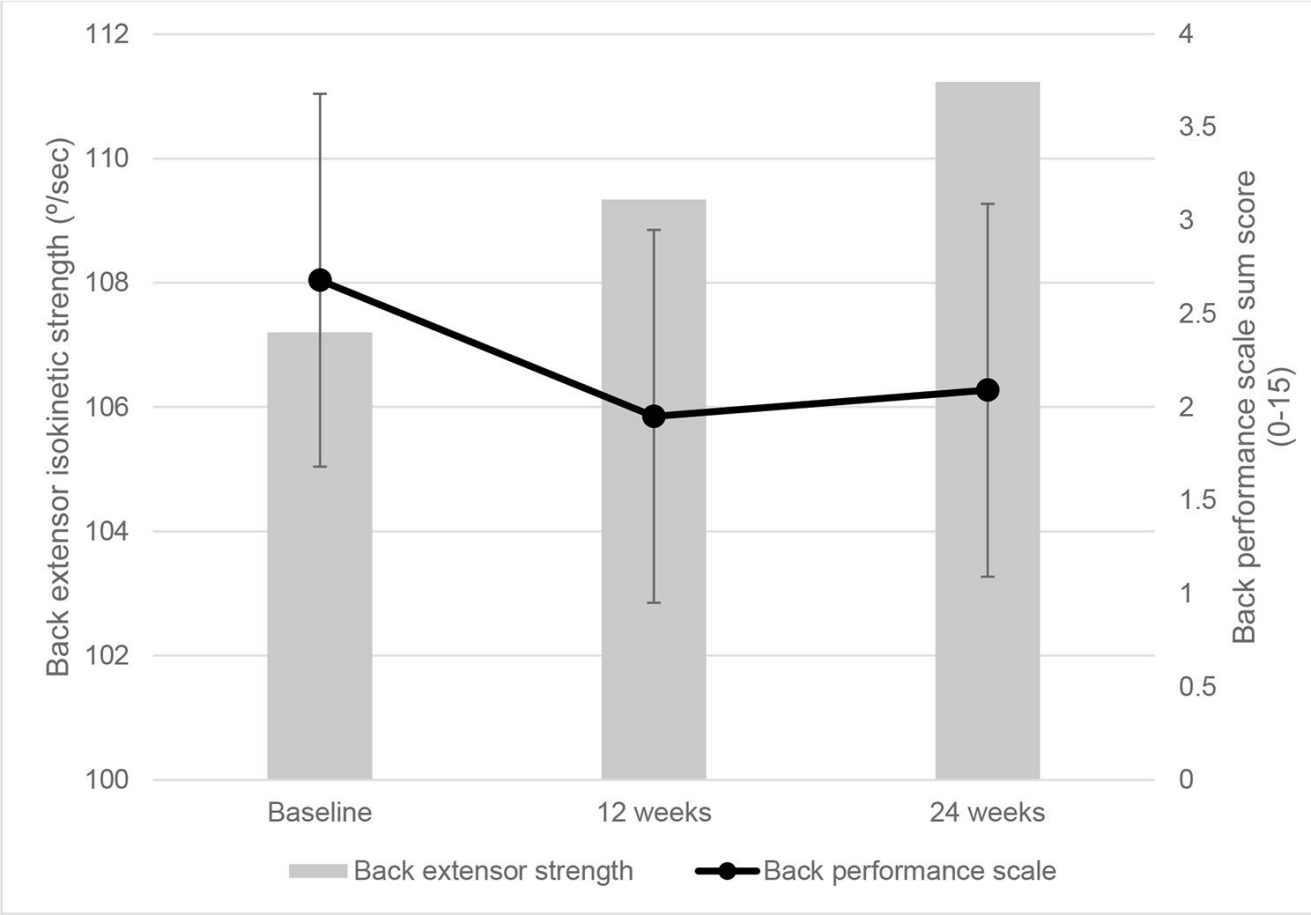
Changes of Back extensor isokinetic strength and Back performance scale sum score

The conventional sarcopenic indices, ASM/Ht^2^, handgrip strength, and gait speed, did not change during the study period. However, SPPB increased from 11.46 ± 0.86 (week 0) to 11.82 ± 0.40 (week 24) (Table 1). Although the LEM mass appeared to increase continuously from 606.14 ± 137.17 (week 0) to 625.05 ± 134.16 (week 12) and 644.18 ± 139.29 (week 24), the changes were not statistically significant (P = 0.107). The LEM volume and SI measured using 3D MRI did not show a significant change. Back extensor strength appeared to gradually increase (107.20 ± 30.66, 109.34 ± 30.88, 111,24 ± 32.8); however, the change was also not statistically significant (P = 0.719). The BPS sum score improved significantly from 2.68 ± 1.81 (week 0) to 1.95 ± 1.21 (week 12) and 2.09 ± 1.34 (week 24) (P = 0.034) (Table 1; Fig. 3).

**Table 1:** Changes of Back extensor isokinetic strength and Back performance scale sum score

	Baseline	12 weeks	24 weeks	P-value*
ASM/Ht^2^ (kg/m^2^)	5.97 ± 0.52	5.97 ± 0.44	6.13 ± 0.60	0.107
Handgrip strength (kg)	22.10 ± 3.42	22.42 ± 2.94	21.95 ± 3.57	0.334
Gait speed (m/sec)	1.253 ± 0.130	1.249 ± 0.126	1.271 ± 0.153	0.303
Short Physical Performance Battery	***11.46 ± 0.86***	***11.77 ± 0.53***	***11.82 ± 0.40‡***	***0.042***
Lumbar extensor muscle mass (g)	606.14 ± 137.17	625.05 ± 134.16	644.18 ± 139.29	0.095
Lumbar extensor muscle volume (cm^3^)	449.12 ± 64.50	446.52 ± 64.19		0.137**
Lumbar extensor muscle signal intensity	287.80 ± 64.42	279.13 ± 78.84		0.363**
Back extensor strength (isokinetic) (º/sec)	107.20 ± 30.66	109.34 ± 30.88	111.24 ± 32.8	0.719
Back performance scale	***2.68 ± 1.81***	***1.95 ± 1.21†***	***2.09 ± 1.34‡***	***0.034***

Abbreviations: ASM/Ht^2^: appendicular skeletal muscle mass/height^2^

Analyzed by *repeated measure ANOVA and **paired t-test

† p < 0.05 by 0 vs. 12 weeks by post-hoc analysis

‡ p < 0.05 by 0 vs. 24 weeks by post-hoc analysis

## Discussion

In this open-label, single-arm prospective study with 26 older women diagnosed with spinal sarcopenia, the combined exercise and nutrition intervention could be feasible and helpful in improving the physical performance as well as back performance. Although the back extensor muscle mass and strength appeared to improve, these trends were not significant. To the best of our knowledge, this is the first clinical study to investigate the effects of exercise and nutritional interventions in older adults with spinal sarcopenia.

Sarcopenia is a multifactorial disorder that is characterized by age-dependent progressive muscle atrophy and fatty degeneration [8]. Since large paraspinal muscles cover the spine and sarcopenic changes of the muscles are associated with spinal dysfunction, spinal sarcopenia has been attracting attention as a spinal disorder associated with chronic back pain often encountered in aging populations [6]. The need for regional paraspinal muscle evaluation has been suggested because conventional sarcopenic indices (appendicular skeletal muscle mass, handgrip strength, and gait speed) are not directly associated with the paraspinal muscles.

Several studies have quantitatively measured the cross-sectional area (CSA) of muscles using imaging techniques such as MRI and CT [6, 24–27]. However, because the paraspinal muscles are among the longest muscles in the human body, the CSA of one part alone might not be able to accurately reflect the overall muscle mass. Furthermore, measuring CSA from MRI or CT scans is neither simple nor feasible in clinical settings. Therefore, we have suggested a quantitative evaluation of the muscles in this region using lateral whole-body DXA [5], which was used to define spinal sarcopenia in this study. The cutoff value of lumbar extensor muscle mass measured by lateral DXA for spinal sarcopenia was set at 562 g as the value of the lower 50th percentile of measurements [12].

Previous studies on exercise and nutritional intervention in patients with sarcopenia have reported varied results regarding the benefits of protein supplementation in addition to exercise alone. Some studies reported additional benefits of nutritional intervention [28], but other studies no additional benefits have been reported [29–31]. The varying effects of protein supplementation observed in previous studies could be attributed to differences in the quantity and quality of protein provided, as well as the presence of other nutrients such as creatine, vitamins, and essential amino acids. In this study, due to the limitations of the study design, which is single-arm, before-and-after study, it is not possible to analyze the effect of nutritional intervention separately. Future large-scale randomized controlled trials may be needed to further investigate the effectiveness of nutritional intervention in patients with spinal sarcopenia. Furthermore, recent studies have reported that elderly patients with sarcopenia may experience malnutrition due to oral frailty and sarcopenic dysphagia [32], and decreased swallowing function has been observed even before the onset of clinical dysphagia [33]. In addition, it would be necessary to consider not only the type and amount of nutrients but also the route and form of intake regarding the nutritional support. Therefore, considering those factors, future studies may need to screen for tailored risk factors to perform a personalized approach.

Although the change was under minimal clinically important difference [9], the SPPB increased from 11.46 ± 0.86 (week 0) to 11.82 ± 0.40 (week 24), while the BPS sum score significantly improved from 2.68 ± 1.81 (week 0) to 1.95 ± 1.21 (week 12); the improvement persisted after the intervention was discontinued (2.09 ± 1.34, week 24). However, the lack of improvement in the lumbar back muscle mass or volume was an unexpected result. Another study has reported that resistance training for patients with sarcopenia resulted in the improvement of their physical performance, such as SPPB; however, skeletal muscle mass did not increase [34]. The authors suggested that the improvement of functionality without muscle mass increase was due to an increase in the motor unit recruitment capacity and motor unit firing rate [35], and muscle hypertrophy occurs after neurological adaptation [36]. In similar results from Stover et al. [37], progressive resistance training twice a week for 16 weeks led to a significant increase in the SPPB score, but not in the skeletal muscle mass index. They interpreted this result as changes in muscle strength and performance might have occurred on the neurophysiological level; however, no explicit measurements were conducted.

The combined exercise and nutrition intervention was not effective in improving back extensor muscle strength, although the strength appeared to gradually increase from baseline (week 0) to follow-up (week 24). Thus, we could interpret that this intervention was sufficient for improving physical performance, but not for increasing the muscle mass or strength. We speculate that physical performance improvement could precede muscle strength increase because muscular functionality is improved by enhancing the activation, firing frequency, and synchrony of motor units. Moreover, muscle quality, such as fiber type, architecture, neuromuscular activation, and aerobic capacity, has recently been linked to muscle function [38]. Frequent exercise training is known to be associated with microscopic changes in skeletal muscles, such as changes in contractile protein, metabolic regulation, intracellular signaling, and mitochondrial activities [36, 39]. Therefore, combined exercise-nutrition intervention-induced microscopic changes affecting muscle quality could improve the physical performance before significantly increasing the muscle strength.

This study has several limitations. First, since our cohort consisted of healthy community-dwelling elderly individuals, the most of them would be engaging in adequate exercise and receiving optimum nutrition in daily life. Therefore, it is possible that the 12 weeks of combined exercise and nutrition intervention were not sufficient to increase the muscle mass in this sample. It is necessary to conduct an interventional study targeting the elderly who are more sarcopenic and frail or have LEM atrophy due to diverse spinal diseases. Second, it was difficult to accurately interpret the variables evaluated at week 24, as there were no restrictions on physical activities during the 12-week follow-up period after the intervention (between week 12 and week 24). For instance, few participants might have continued exercising during the follow-up period, whereas others might have returned to their previous lifestyle with no exercise. In future research, these factors should be controlled. Third, the diagnostic criterion for spinal sarcopenia in our study was adopted arbitrarily as a lower 50th percentile of LEM mass of our cohort. Furthermore, we choose back extensor muscle strength as the primary outcome measure, rather than LEM mass, to observe changes in muscle quality as the intervention of this study focused on resistance training and nutritional supplementation. This discrepancy may be attributed to the fact that diagnostic criteria for spinal sarcopenia have not yet been established and may require the development of diagnostic algorithms like those used for classical sarcopenia in future studies. Finally, the small study population (N = 26) may limit the generalizability of the results. There are few references for the standard deviation and effect size differences in back extensor muscle strength among the elderly population, which is the primary outcome variable. Consequently, the sample size calculation was not feasible. Therefore, we conducted the study on a feasible sample within our cohort. Large-scale randomized controlled trials are needed to confirm the findings of this study.

In conclusion, the combined resistance exercise and nutritional intervention for community-dwelling older women with spinal sarcopenia could be feasible and helpful in improving their physical performance. Further long-term, controlled trials are required to validate this combined intervention.

## Data Availability

The datasets used and analyzed during the current study are available from the corresponding author on reasonable request.

## Abbreviations

ASM/Ht^2^: appendicular skeletal muscle mass/height^2^
BPS: back performance scale
CSA: cross-sectional area
CT: computed tomography
DXA: dual-energy X-ray absorptiometry
LEM: lumbar extensor muscle
MRI: magnetic resonance imaging
